# Environmental Assessment of a Hybrid Solar-Biomass Energy Supplying System: A Case Study

**DOI:** 10.3390/ijerph16122222

**Published:** 2019-06-24

**Authors:** Congguang Zhang, Jiaming Sun, Jieying Ma, Fuqing Xu, Ling Qiu

**Affiliations:** 1 College of Mechanical and Electronic Engineering, Northwest A&F University, Yangling 712100, China; zhang.10047@osu.edu (C.Z.); cn734057@gmail.com (J.S.); Jeremy_lee1@163.com (J.M.); 2 Western Scientific Observation and Experiment Station of Development and Utilization of Rural Renewable Energy of Ministry of Agriculture, Northwest A&F University, Yangling 712100, China; 3 Department of Food, Agricultural and Biological Engineering, The Ohio State University, Wooster, OH 44691, USA; xufuqing@xjtu.edu.cn; 4 School of Human Settlements and Civil Engineering, Xi’an Jiaotong University, Xi’an 710049, China

**Keywords:** anaerobic digestion, solar energy, life-cycle assessment, environmental emissions, energy consumption

## Abstract

Local energy supply by renewable energy, such as solar energy and biomass, using distributed energy systems plays an important role in global energy structure. This study investigated the environmental performance of a hybrid solar-biomass energy supplying system by life-cycle assessment method. The results showed that in terms of environmental and energy impacts, the construction stage and the disassembly and recycling stage of the system contribute little to the whole life-cycle environmental impacts. According to the results of most of the selected impact categories, the solar subsystem contributed the most environmental emissions during construction stage, followed by the two anaerobic reactors; therefore, the excessive pursuit of high solar energy proportion can correspondingly lead to even more serious environmental problems. The integrated energy supplying system significantly reduces non-renewable energy consumption, climate change impacts, acidification as well as eutrophication effects due to the replacement of alternatives such as lignite coal, and from fertilizer production. The present hybrid solar-biomass energy supplying system not only produces clean thermal energy but also reduces the disposal of organic wastes and produces valuable agricultural products.

## 1. Introduction

For a long period of time, especially since the launch of the Industrial Structure Optimization and Upgrading Plan in recent years, the adjustment of energy structure is an essential new and higher requirement for renewable energy in China. For instance, the country’s target for Intended Nationally Determined Contributions (INDC) published in 2015 demonstrated that the renewable energy development plan will be a key part in China’s low-carbon economy [1]. Not only in China, energy and climate policies around the world have greatly encouraged the development of energy production from renewable sources, such as biomass, wind, solar, hydro-power, and geothermal, which can provide sustainable energy services based on the utilization of routinely available indigenous resources [2,3].

In recent years, there have been quite a few projects that have applied the hybridization of different renewable energy technologies to further promote sustainable development and to seek better energy supply system. Hybrid energy systems are usually presented as a viable, safe and effective solution to minimize the dependence on a single renewable resource, which is particularly important in areas with scarce natural resources and unstable supplies [4]. According to Semaoui et al., the incorporation of renewable energy sources can be a non-polluting solution for power generation, allowing distributed supply of energy in the geography of a country or district, as well as providing a viable alternative for isolated generation applications [5,6].

Among these hybrid systems, the hybrid solar-biomass system is regarded as a generally satisfactory mode based on a series of thermodynamic, economic, and environmental evaluation results [7,8,9]. Generally, the solar-biomass systems can make full use of local biomass resources and solar radiation resources, local energy structure can be improved by using such renewable energy systems and some of the environmental problems like frequent haze may be solved effectively. It should be noted that most of the hybrid solar-biomass systems are focusing on drying [10], power generation [11,12], biomass gasification [13] or multi-generation [9,14,15], only a few systems are focusing on house energy supplying [16,17]. Even among hybrid solar-biomass energy systems, most biomass subsystems directly burn agricultural residues or forestry biomass. There were few reports on anaerobic digestion subsystem, which had been proved as a negative “net carbon emission” bioenergy technology [18]. Biomass is regarded as a kind of carbon-neutral energy resource and, in theory, burning biomass is considered carbon neutral because it is only releasing carbon dioxide that was captured in the first place when the biomass plant was growing, so the capture of most combustion emissions creates a net negative emission across the bioenergy utilization. From this point of view, if the greenhouse gas can be controlled effectively during the anaerobic digestion process, the biomass subsystem can be regarded as a “net negative-emission” system [19].

LCA is used to evaluate the environmental impacts associated with the entire life-cycle of a product, process, or activity. It has been widely used for eco-labeling programs, strategic planning, and marketing [20]. In recent years, there have been plenty of studies on the LCA of single biogas systems or single solar systems [21,22]. However, most of them are mainly based on technical elements, and almost no report has been found that focuses on the construction of the above system. This is mainly because some scholars believe that when the lifetime of a facility is considered, generally the related environmental emissions released by the facility only represented <2% of the annual operational emissions, and are thus often ignored [23,24]. However, through the environmental analysis of system construction, a better understanding of the system feature will be obtained, especially for hybrid energy systems. As is already known, the construction scale of different subsystems affects their proportion in the whole system. Through the analysis of system construction, the environmental sustainability of different subsystems can be predicted from the perspective of the main functional modules of the system, and the system configuration can also be optimized to improve the overall performance of the system.

Therefore, for energy systems, it is meaningful to quantify and analyze the environmental emissions of the construction, which can thus avoid excessive capital investments on part of the system and thus ignore possible environmental problems. It also allows us to make decisions according to local circumstances. For instance, allocating more solar energy in places where it is abundant, and decreasing the proportion of bioenergy where biomass is insufficient. The significant characteristics of a hybrid energy system are to combine two or more renewable energy generation technologies to make proper use of their operating characteristics and to obtain efficiencies higher than that could be obtained from a single energy source. [25] Generally, LCA of these hybrid energy systems can provide important reference and data for the optimization of the system, and the improvement of system efficiency as well as the selection of equipment and system structure. Moreover, as a hybrid energy system, there is also an important issue that the balance of different energy sources of the system should be investigated in order to optimize the environmental and/or economic performance, for example, Ogunjuyigbe et al. studied the optimal allocation and sizing of a PV/Wind/Split-diesel/Battery hybrid energy system for minimizing life-cycle cost, carbon emission and dump energy of remote residential building [26], Nojavan et al. optimized a battery/PV/fuel cell/grid hybrid energy system using information gap decision theory [27].

According to the system boundary, LCA can be divided into consequential LCA and attributional LCA. Consequential LCA provides detailed information about the consequences of changes in the level of output (consumption and disposal) of a product or a system, including effects both inside and outside the life cycle of the product or system [28]. For hybrid energy systems especially the renewable energy systems, it is vital to understand and quantify the environmental benefits generated from the utilization of renewable resources as well as the corresponding resources being replaced. Through the LCA of the hybrid solar-biomass energy system, the stakeholders and policymakers can obtain a more comprehensive understanding of these systems and explore more details about the practical feasibility of these engineering projects. According to the LCA study of a solar-assisted hybrid CCHP system designed by Wang et al., to achieve the optimal performance of the energy system, LCA optimization is an efficient methodology to configure the hybrid CCHP capacity and optimize operation strategy. Moreover, the economic optimization may also be achieved by LCA method, for example Gan et al. analyzed the life-cycle cost of a hybrid wind–photovoltaic–diesel–battery system in Scotland [29], and Lajunen et al. conducted a life-cycle cost assessment of diesel, natural gas, hybrid electric, fuel cell hybrid and electric transit buses [30]. However, in more cases, LCA is considered to be an important tool to measure the environmental benefits of a renewable energy system [31,32].

The primary objective of this study was to assess the environmental impact of the designed energy supplying system and to describe how the environmental and energy performance is affected by the different system stages, as well as exploring the major sources of net environmental emissions during the system construction. Therefore, primary energy demand, material input and output, global warming potential, as well as acidification potential of the system were evaluated through LCA.

## 2. Materials and Methods

### 2.1. Case Study

Chicken manure was obtained from the Third Farm of Northwest A&F University, Shaanxi, China. After removing the debris, the chicken manure was crushed and mixed into homogenized matrix using a blender then stored in a refrigerator at 4 °C before use. Activated sludge obtained from thermophilic AD was used as inoculum, which was collected from the Fifth Sewage Treatment Plant in Xi’an, Shaanxi. The physio-chemical characteristics of the chicken manure and inoculum are summarized in Table 1. The hybrid solar-biomass energy supplying system was designed and fabricated at Western Scientific Observing and Experimental Station for Development and Utilization of Rural Renewable Energy, Ministry of Agriculture (34.31° N, 108.06° E), Yangling, China. Based on the abundant solar and biomass resources in the northwest areas of China, as well as the strong demand for energy structure optimization and adjustment in these areas, it would be even more practically significant to explore the feasibility of spreading hybrid solar-biomass energy development and utilization mode.

**Figure 1:**
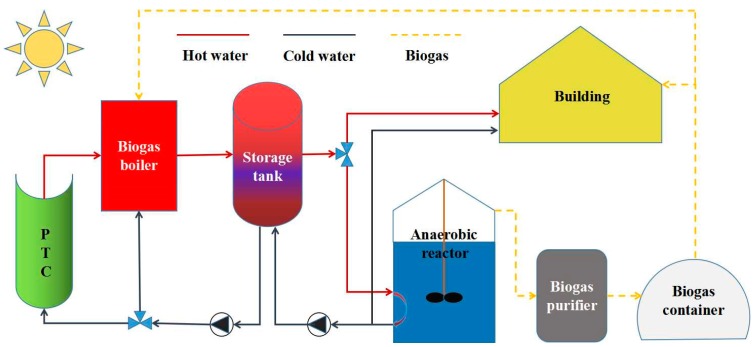
Schematic diagram of the hybrid solar-biomass district heating system.

In detail, as shown in Figure 1, the system consists of a solar subsystem, a biogas subsystem, control units, piping and duct, pumps and valves, as well as some necessary heating appliances. The biogas subsystem is mainly comprised of a 75-m^3^ continuously stirred tank reactor (CSTR) and a 75-m^3^ up-flow blanket filter (UBF). Key parameters of the biogas subsystem are listed in Table 1. As for the solar subsystem, according to thermodynamic and life-cycle assessment of typical solar systems such as the solar photovoltaic system and the parabolic trough solar collector system, the latter one shows favorable sustainability and feasibility in rural areas [33]. Therefore, to maximize the use of solar energy and to minimize the environmental emissions, this study chose the parabolic trough collector (PTC) as the main functional component of the solar subsystem. For the “biomass part”, although the way biomass direct combustion may bring considerable economic benefits, the carbon-negative energy conversion path from biomass to biogas can be more environmentally sustainable in terms of avoiding direct emissions of certain harmful organisms from waste biomass, as well as producing nutrient-rich organic fertilizer [21]. The feedstock used for anaerobic digestion is swine manure collected from an adjacent local farm. It is assumed that the transportation of feedstock from the farm to the biogas subsystem can be neglected, but the utilization of biogas slurry and digestate will consume transportation fuel.

### 2.2. Life-Cycle Assessment

LCA is a methodological framework useful and powerful to evaluate the environmental impacts of a system, product or activity [34]. The evaluation includes the entire life cycle of the product, process, activity or a complex system, from cradle to grave [35]. LCA has been selected for the environmental analysis performed in this study. Generally, the implement of LCA can be divided into several key steps, such as goal and scope definition, functional unit determination, and life-cycle inventory analysis.

#### 2.2.1. Goal and Scope Definition

**Figure 2:**
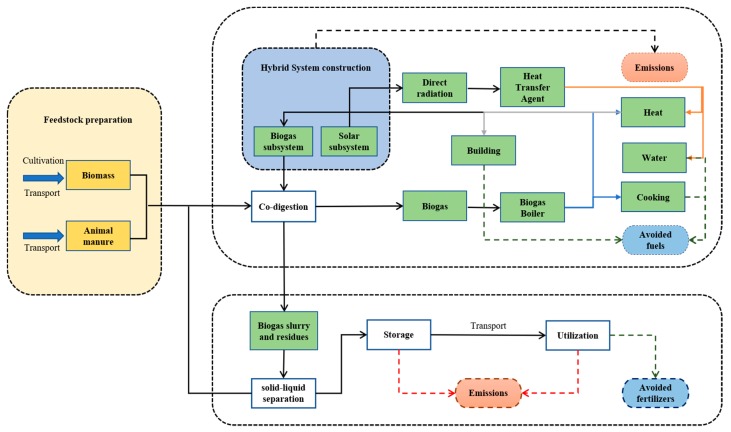
System boundary.

The goal of this LCA was to use a cradle-to-grave approach to evaluate the environmental performance of a thermal energy supply to a residential building for heating and domestic hot water using a novel solar-biomass energy supplying system. Like common energy systems, the energy produced by the current solar-biomass energy supplying system is distributed through a district heating network that connects the energy production plant to the neighborhoods and delivers the energy to individual buildings [36]. Figure 2 shows the system boundary of the present LCA, as well as the reference flows. As can be seen, a portion of the heat energy is distributed to different sectors of the system based on different seasons or facilities. An interesting thing to note is that the LCA conducted in this study can be regarded as a consequential LCA, in which the utilization of system products including biogas and digestate are considered as a substitution of organic fertilizer. Like most previous LCA studies, the present LCA also considered that the biogas utilization replaces the burning of lignite, which is a common energy source in the local area. Similarly, organic fertilizer was substituted through the utilization of digestate. Therefore, the substitution of materials is included into the system boundary.

#### 2.2.2. Functional Unit

According to ISO standards, the functional unit (FU) is defined as the main function of the system expressed in quantitative terms [34]. The main function of the present hybrid solar-biomass energy supplying system is the anaerobic digestion of feedstock for biogas production as well as collecting solar irradiation through a PTC in order to cogenerate energy. Although the solar energy fluctuates severely along the year, the present system can adjust the ratio of solar energy and bioenergy according to the season thus, under the premise of satisfying the heating demand of the building, uses as much solar radiation as possible. Meanwhile, potential environmental emissions from the manufacture of solar equipment are also considered.

Based on the above energy changes, to be convenient, the calculations and analyses were based on each year. The area of the solar collector was 48 m^2^ with a solar fraction of 34.4%. The thermal power of the biogas boiler was 40 kW. The maximum daily biogas consumption of the system was 127.5 m^3^ and the daily biogas production was approximately 240 m^3^. According to the thermodynamic analysis, the heat consumption of the building was calculated as 787.5 MJ/d. the total heat load of the biogas subsystem was 274.47 GJ/year, including the energy consumption for heating the feedstock mixture (110.12 GJ/year) and the heat loss of the anaerobic digesters (164.34 GJ/year). The total heat output of the biogas boiler was 355.69 GJ/year while the cumulative heat output of the solar subsystem was 89.86 GJ/year, hence the system can operate smoothly without external energy supply. Therefore, based on detailed calculation, the FU was chosen to be 1000 GJ thermal energy produced by the proposed energy supply system.

#### 2.2.3. Data Source and Assessment Indicators

The environmental profile was estimated by using the characterization factors reported by the CML (characterization methods of LCA) baseline 2001 impact assessment method. Just like with most previous LCA studies upon renewable energy systems or district heating systems, at midpoint level, four commonly used environmental impact indicators were selected in this study, including global warming potential (GWP) expressed as CO_2_ equivalents; primary energy depletion (PED) expressed with the unit MJ; acidification potential (AP) expressed as sulfur dioxide equivalents (SO_2_ eq); and eutrophication potential (EP) expressed as phosphate equivalent (PO_4_^3−^ eq) [37,38].

Data collection was based on site inspections (the Western Scientific Observatory Experimental Station for the Development and Utilization of Rural Renewable Energy), the National Emission Standards of pollutants, the National Bureau of Statistics of China [39]. The LCA model was established in eFootprint software (IKE Environmental Technology Co., Ltd., Chengdu, China) with Chinese LCA database (CLCD) and Ecoinvent 3.0 database. The anaerobic digestion feedstock used in this study was collected from a nearby farm (less than 5 km from the biogas plant). Original meteorological data of local renewable resources (including solar radiation, wind, and rainfall) are derived from the China Meteorological Data Sharing Service System (http://www.cma.gov.cn/).

### 2.3. Life-Cycle Inventory Analysis

For previous LCA studies of industrial systems, most of them can ultimately be divided into three stages, i.e., construction stage, operation stage, and abandonment stage. It is worth mentioning that comparing different LCA results from previous studies may be misleading as estimates may vary significantly depending on the system boundaries, assumptions, models and even database used [40]. However, comparing different scenarios or subsystems within one study (with the same assumptions, input data and models) may lead to reliable results that can provide valuable reference to policy makers [41]. Therefore, the hybrid solar-biomass energy supplying system was divided into system construction stage, system operation and maintenance stage, as well as system disassembling and recycling stage in this study.

#### 2.3.1. System Construction Stage

As mentioned above, the overall system can be simply regarded as the combination of a solar subsystem and a biogas subsystem. According to previous studies [42,43], a biogas plant generally constitutes a digestate storage tank, a digester tank, a heat exchanger unit, piping/pumps, valves, motors, etc. Therefore, the construction of the analyzed biogas subsystem basically included the above subassemblies. The solar subsystem mainly included a PTC and a series of auxiliary modules, e.g., the bracket and stakes. Based on the detailed calculations and for the convenience of analyzing, we divided the whole system into six modules in total, i.e., a CSTR, a UBF, an integrated secondary fermentation tank (ISFT), a blending/storage tank (BST), a solar heating module (SHM) and a set of ancillary works (AW).

Each module consists of various materials, for example, the CSTR module has concrete, water-proofing additive, rebar, fixed bolt, steel nail, and various steel pipes, etc. Since energy and fuels are important to the system, all the electricity and diesel consumptions are regarded as ancillary works in this study. The hybrid solar-biomass energy supplying system is designed and constructed in northwest China (specifically, Shaanxi Province), and the calculation of the environmental emissions is based on the CLCD database, so the environmental impacts caused by electricity consumption are calculated entirely based on a special data set (Northwest Power Grid Power Mix (China)) inside the software. The data set represents the industry statistical average, which internally includes coal-fired power generation, hydro-power generation, wind-power generation as well as the power mixing and transmission. It should be noted that China’s power grid has calculated the coal consumption for power generation and has already deducted coal consumption for heating.

#### 2.3.2. System Operation and Maintenance Stage

In this study, we mainly focused on the energy supply system, thus, the livestock breeding is not considered as a part of feedstock preparation process. Instead, only transportation was included. In the system operation and maintenance stage, the direct inputs into the system were the feedstock, solar irradiation, the electricity use within the plant, and the heat energy required to heat the anaerobic digesters. Indirect inputs included the energy consumed in the related machinery. During the use of produced biogas, the low-pressure gas was burned in a low NO_x_ condensing, non-modulating boiler to provide energy for residents. Existing inventories within the Ecoinvent 3.0 database were modified to replace the local frequently used coal with upgraded biogas. Since the present LCA is a consequential LCA, it was assumed that the combustion of biogas in a condensing boiler offset the combustion of lignite coal. This substitution therefore included the material flows and emissions associated with the production and transportation of lignite coal and the combustion of lignite coal at end use. A total quantity of 183.67 kg coal is substituted when producing per FU of heat energy.

The anaerobic digestion feedstock comes from a livestock farm near the system, based on which the transportation of a feedstock (22.93 t/day of swine manure with a moisture content of 75.53%) was neglected. However, the digestate was treated through a solid-liquid separator, 22.51 t/d of the liquid digestate was sent to a local farm (Yangling Modern Agricultural Innovation Park). The distance between the hybrid solar-biomass system and the farm is 3.8 km and 4.81 t/d of solid digestate was sent to the farm every day. The digestate is finally used as organic fertilizer, thus can substitute a good deal of chemical fertilizer. According to the investigation, 79.06, 5.21, 38.88 and 3.91 t of nitrogen, phosphorus, potassium, and magnesium fertilizers were substituted, respectively.

#### 2.3.3. System Disassembling and Recycling Stage

In this stage, it was assumed that 90% of the available construction materials can be recycled and further utilized. These materials, including glass, bracket and rebar, were sent to a local hardware factory by truck. Most of the other materials, such as the waste UPVC (unplasticised polyvinyl chloride), PPR (polypropylene-random) and PE (polyethylene) tubular products, were transported to the nearest landfill.

## 3. Results and Discussion

### 3.1. Comparison of Different Life-Cycle Stages

#### 3.1.1. System Construction Stage

**Figure 3:**
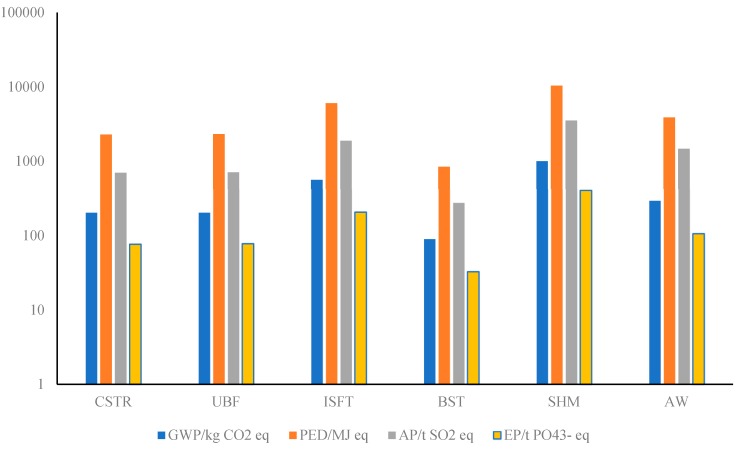
GWP, PED, AP and EP equivalents of the six system modules. Note: GWP—global warming potential, PED—primary energy depletion, AP—acidification potential, EP—eutrophication potential.

Figure 3 summarizes the LCA results obtained when comparing the main function modules that jointly make up the system. Because of the large-scale difference between different impact results, the vertical axis is divided logarithmically. As for the energy production modules, CSTR, UBF and SHM are the most important to the system, and the total environmental impacts of them accounted for 55.73% (GWP), 58.37% (PED), 57.64% (AP) and 61.92% (EP), respectively, of the whole system. In other words, they accounted for most of the system environmental emissions during the system construction phase.

Among the six modules in system construction stage, solar heating module (SHM) is one of the energy supply parts, it had the most significant effect towards the system compared to the other five modules, as shown in Figure 3. For example, the PED of the SHM accounted for approximately 40.47% of the total system energy consumption. Therefore, although some scholars suggest that the proportion of solar energy in the hybrid energy systems should be increased as much as possible because of the lower price of solar source, its proportion still needs to be reasonably regulated based on the performance assessment of environmental factors. Among the main sources of environmental emissions in solar subsystem, the data showed that most emissions originated from the production of stakes, which respectively accounted for 91.70% (GWP), 88.99% (PED), 84.17% (AP) and 82.27% (EP) of the total impact values of the system. In contrast, as the main components of the SHM module, the glass reflectors and brackets of the PTC lead to less than 20% of the impact value of every environmental indicator, for example, they accounted for only 8.14% of GWP. Therefore, it was found that the primary sources of the solar system emissions were not the reflector on the collectors, but the fixed stakes between the ground and the reflector.

**Figure 4:**
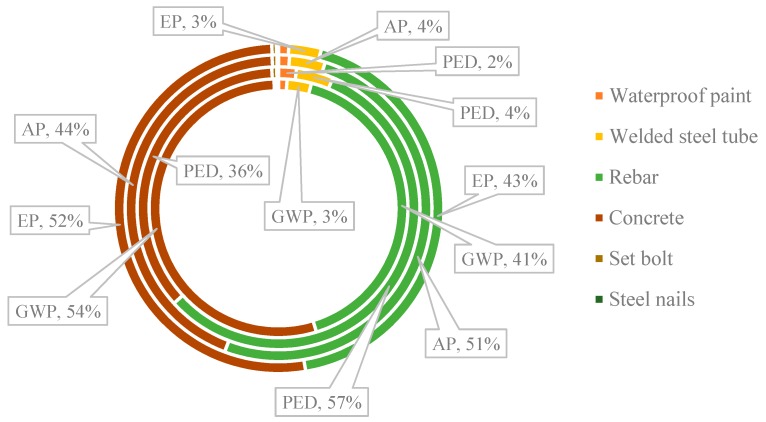
Contributions of main materials to the environmental burden of the ISFT (integrated secondary fermentation tank) module (only items contributed large than 1% are included). Note: GWP—global warming potential, PED—primary energy depletion, AP—acidification potential, EP—eutrophication potential.

In terms of the comprehensive environmental impact, as represented in Figure 3, the emission sources next to the SHM is ISFT based on the results of all indicators. According to Figure 4, the most significant sources of environmental impacts were concrete and steel in terms of the ISFT module, they accounted for 54.17% and 41.16% of GWP respectively. According to the calculation, for all equivalents of the four impact indicators, the sum of concrete and steel accounted for more than 90% of each of these indicators. This showed that infrastructure construction related materials occupied a high position in the system construction stage, and these materials were almost inevitable and difficult to optimize in most cases. Moreover, the influence of welded steel pipe and waterproof paint was small (see Figure 4). In this study, only the preparation of acrylic acid and vinyl acetate was considered for the calculation of waterproof paint.

Because the design specification is basically the same for the two anaerobic digestors, i.e., CSTR and UBF, there was no significant difference between their environmental impacts. Taking the environmental performance of CSTR as an example, like the ISFT, the most significant emissions were due to the raw materials including concrete and steel. However, due to the different production processes of the two, the impact on each indicator was slightly different. For instance, as for GWP and EP, the influence of concrete was greater than steel, however, the result was exactly the opposite in terms of PED and AP.

According to the life-cycle inventory analysis, AW also had a large impact to the environment. In this module, power consumption had the highest contribution (more than 80% for each impact category) to the environmental emissions and energy depletion, which may be because the electricity required during the system construction was classified into the AW module. The electricity consumed was mainly used for external machinery and equipment including pumps and blenders, etc. The remaining emissions and energy consumptions mainly came from various pipe materials involved in the system, such as steel pipes (internal and external of the anaerobic reactors), PVC and UPVC pipes connecting each unit. UPVC pipes were responsible for 7.40–10.08% of each impact category.

According to the results of impact from GWP, PED, AP and EP, the environmental contribution of BST was mainly derived from concrete (between 55.74–73.13%), followed by steel (24.25–38.41%) and waterproof paint (2.57–5.85%). From this point of view, concrete and steel, as the most commonly used infrastructure materials, have a great impact on the environment during the system construction stage, which is in line with our common sense.

#### 3.1.2. System Operation and Maintenance Stage

**Figure 5:**
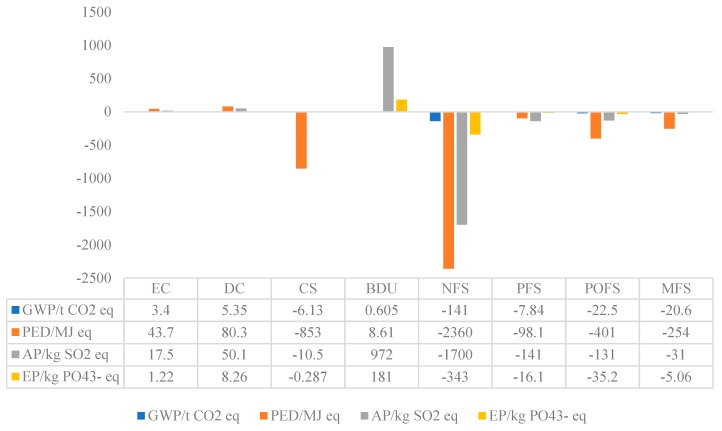
Primary GWP, PED, AP and EP equivalents of the system operation and maintenance stage.

In this stage, the hybrid solar-biomass energy supplying system provides thermal energy in the form of hot water for the nearby building. Based on the system characteristics and key components, we considered the following parts as the life-cycle inventory of the system operation and maintenance stage: electricity consumption (EC), diesel consumption (DC), coal substitution (CS), biogas digestate utilization (BDU), nitrogen fertilizer substitution (NFS), phosphate fertilizer substitution (PFS), potassium fertilizer substitution (POFS) and magnesium fertilizer substitution (MFS). As shown in Figure 5 that positive values are indicative of environmental burdens whereas negative values signify environmental credits or benefits accrued from carbon dioxide uptake and the substitution of mineral fertilizer and lignite.

To produce each FU of thermal energy, a total equivalent quantity of 9355 kg of CO_2_ will be released. The contribution of system operation and maintenance stage to total greenhouse gas emissions is due to DC (57.19%), EC (36.34%) and BDU (6.47%), therefore it can be found that the external energy consumption during system operation processes have the highest influence on GWP. The PED contribution of external energy consumption is equal to 13.26 MJ: 60.55% is due to the use of external electricity and the 32.95% is due to diesel consumption for transportation.

Biogas digestate utilization (BDU) accounted for the highest proportion of AP (972 kg SO_2_ eq) and EP (181 kg PO_4_^3−^ eq), this may be due to the high NH_3_ and phosphate field emissions along with the use of digestate. According to Sommer and Hutchings and Ramírez Arpide et al., field applied organic matter contributes significantly to the emission of NH_3_ from agriculture [44]. In contrast, the full use of digestate substituted a large quantity of chemical fertilizers, the substitution consists of NFS, NFS, PFS and MFS, and all their values are negative while the NFS contributed most of the avoided environmental burden.

Overall, the system operation and maintenance stage showed a negative impact on the four selected impact categories. As we know, for most bioenergy production methods, the net negative emissions were mainly due to the potential CO_2_ emissions sequestered from the organic matter. The CO_2_ fixation was accounted for as a consumption of the CO_2_ resource in the production processes of biogas, it was assumed that CO_2_ was firstly consumed to generate the anaerobic digestion feedstock (animal feedstock preparation in this study) and therefore was required within the biogas subsystem [39]. However, in this case, as described in Figure 5, it is more important to note that the substitution of coal and chemical fertilizer explained most of the negative CO_2_ emissions.

#### 3.1.3. System Disassembling and Recycling Stage

Generally, the specific processes of the system disassembling and recycling can vary dramatically based on different criteria. In this study, according to the impact factors such as the resource availability and the significance to the overall system, we considered five processes including rebar recycling, bracket recycling, glass recycling, wastes landfill, and transportation. The recycling of unspoiled construction materials can be regarded as an avoided process to produce these materials; thus, the values of all impact categories should be negative. The upstream emission factors are directly derived from the Ecoinvent 3.0 database. As can be seen from Table 2, compared to the environmental impacts of transportation and landfilling of system wastes, for each FU of energy production, the recycling of rebar, bracket and glass is almost negligible. Transportation process is the item contributing more than 80% of environmental emissions in all impact categories.

### 3.2. Overall Environmental Evaluation

In the above sections, the life-cycle impact analysis of each stage is described in detail. As an integrated energy system, from a long-term point of view, broader environmental analysis of the present hybrid solar-biomass energy supplying system should be emphasized. Table 3 shows the total life-cycle impact assessment results for the three system stages. The results are discussed below by impact categories, focusing on the comparison between different stages as well as finding out the unit life-cycle process that generated important impacts to the whole system.

According to the results, it can be found that the system operation and maintenance stage shows the highest influence over the environmental profile, especially in terms of AP and EP, which contributed more than 90% of the emissions (only total impacts are included). On each day of the system operation, there are a plenty of resources getting in and out of the system, especially for the anaerobic digestion process. In this process, for each functional unit of energy production (1000 GJ), a quantity of 3221.54 t of digestate would be utilized in local field, and 21,400 t·km of diesel as well as 3760 kWh of electricity would be consumed for transportation and equipment operation. During the system operating period, electricity consumption and diesel consumption are the two most prominent factors affecting the environment, it is easy to calculate that the system consumes 9540 t*km of diesel as well as 1675.64 kWh of electricity each year, which are much higher than that in the construction period. Therefore, from this point of view, it can be seen that strictly controlling the external energy consumption in the operation stage of the system has the feasibility of significantly improving the economic performance and reducing the environmental emissions effects.

The results showed that the utilization of the end products (e.g., digestate and biogas) can help reduce greenhouse gas emissions as well as evidently lower the primary energy consumption, acidification, and eutrophication potentials. However, due to the large amounts of greenhouse gas released from energy use and materials input (Figure 3), the GWP emission of the construction stage (2360 kg CO_2_ eq) was quite significant. Among the six main modules, the total primary energy consumption of the solar subsystem was 10,400 MJ, comprised the largest share (40.31%) of primary energy input during the system construction period. This indicates that the solar energy proportion is not the higher the better in northwest China, but there should be an optimal level depending on the location. In terms of GWP, the solar heating module contributed the most to the greenhouse gas emissions (1000 kg CO_2_ eq), followed by the UBF (205 kg CO_2_ eq) and the CSTR (202 kg CO_2_ eq).

It can also be found that the system construction stage contributed much more environmental impacts than the system disassembling and recycling stage (Table 3), which is in line with the actual production practice. As an example, the GWP of the system construction stage is 28.71 times of that of the system disassembling and recycling stage. Furthermore, the system disassembling and recycling stage supplied an overall positive impact reduction due to the recycling of construction materials that can continue to be used in the future.

## 4. Conclusions

This study investigated a hybrid energy supply system driven by biomass and solar energy in northwest China. Based on the results obtained, it is obvious that the system construction stage contributes the most to environmental impacts compared to the operation stage and the disassembly stage. To reduce the environmental influence, it is significant to design the energy structure of the system, i.e., to adjust the proportion of solar energy and biomass energy supply according to local conditions. As is well known, the recycling of nutrients from digestate to local farms as fertilizer could create favorable benefits. Using livestock manure in the anaerobic digestion subsystem is advantageous in terms of AP and EP, and meanwhile the greenhouse gas emissions from manure storage could be avoided. Analyzing the overall system process (construction, operation and disassembly), as well as all the unit processes of the system, allowed the users to select optimal processes that minimize the environmental impacts. For example, the feedstock pretreatment including drying or compressing may reduce the emissions originated from transportation. According to the analyses, the hybrid solar-biomass energy supplying pattern is a promising solution to optimize the energy structure and promote the harmonious development of regional socioeconomic in northwest China, where the solar radiation and bioresources are relatively abundant, and corresponding incentive policies should be provided to encourage the wide spread of the proposed energy supplying mode.

## Figures and Tables

**Table 1:** Characterizations of the biogas subsystem and feedstock.

Items	Unit	Pig Manure
HRT	d	20
Feedstock decomposition	% DM	24.47
	% ODM	17.29
Methane	Vol.% CH_4_	55
Methane yield	m^3^/ t VS	350
Biogas yield	m^3^/d	240
Digestate (solid)	t/d	4.81
Digestate (liquid)	t/d	62.51
Recycled slurry	t/d	40
Concentration of system	% TS	8

Note: HRT = Hydraulic retention period; CH_4_ = methane; TS = total solids; Vol. = volume; DM = dry matter; ODM = organic dry matter (volatile solids).

**Table 2:** Life-cycle impact results of the system disassembling and recycling stage.

	GWP/kg CO_2_ eq	PED/MJ eq	AP/kg SO_2_ eq	EP/kg PO_4_^3−^ eq
Rebar recycling	−5.10 × 10^−2^	−0.754	−2.11 × 10^−4^	−1.98 × 10^−5^
Bracket recycling	−2.71 × 10^−2^	−0.397	−1.06 × 10^−4^	−1.46 × 10^−5^
Glass recycling	−8.12 × 10^−4^	−9.36 × 10^−3^	−1.26× 10^−5^	−1.18 × 10^−6^
Wastes landfill	2.57	3.39	1.22 × 10^−3^	3.37 × 10^−2^
Transportation	79.6	1560	0.829	0.138
Total	82.1	1560	0.830	0.172

eq: equivalent.

**Table 3:** Life-cycle impact results of the overall system.

Stage	Item	GWP/kg CO_2_ eq	PED/MJ eq	AP/kg SO_2_ eq	EP/kg PO_4_^3−^ eq
System construction	Total impact	2360	25,800	8.57	0.91
	Total impact reduction	0	0	0	0
	Net impact	2360	25,800	8.57	0.91
System operation and maintenance	Total impact	9390	133,000	1040	190
	Total impact reduction	−198,000	−3,960,000	−2010	399
	Net impact	−188,000	−3,830,000	−975	−209
System disassembling and recycling	Total impact	82.2	1570	0.83	0.17
	Total impact reduction	−0.08	−1.16	0	0
	Net impact	82.1	1560	0.83	0.17
Lifespan	Total impact	11,800	160,000	1050	191
	Total impact reduction	−198,000	−3,960,000	−2010	399
	Net impact	−186,000	−3,800,000	−966	−208

eq: equivalent.

## References

[1] Wang B., Wang Q., Wei Y.M., Li Z.P. (2018). Role of renewable energy in China’s energy security and climate change mitigation: An index decomposition analysis. Renew. Sustain. Energy Rev..

[2] Devabhaktuni V., Alam M., Depuru S.S.S.R., Green R.C., Nims D., Near C. (2013). Solar energy: Trends and enabling technologies. Renew. Sustain. Energy Rev..

[3] Scarlat N., Dallemand J.-F., Fahl F. (2018). Biogas: Developments and perspectives in Europe. Renew. Energy.

[4] Vivas F.J., Heras A.D.L., Segura F., Andújar J.M. (2018). A review of energy management strategies for renewable hybrid energy systems with hydrogen backup. Renew. Sustain. Energy Rev..

[5] Semaoui S., Arab A.H., Bacha S., Azoui B. (2013). The new strategy of energy management for a photovoltaic system without extra intended for remote-housing. Sol. Energy.

[6] Robitaille M., Agbossou K., Doumbia M. Modeling of an islanding protection method for a hybrid renewable distributed generator. Proceedings of the Canadian Conference on Electrical and Computer Engineering.

[7] Zhang X., Yang J., Zhao X. (2018). Optimal study of the rural house space heating systems employing the AHP and FCE methods. Energy.

[8] Bet Sarkis R., Zare V. (2018). Proposal and analysis of two novel integrated configurations for hybrid solar-biomass power generation systems: Thermodynamic and economic evaluation. Energy Convers. Manag..

[9] Sahoo U., Kumar R., Singh S.K., Tripathi A.K. (2018). Energy, exergy, economic analysis and optimization of polygeneration hybrid solar-biomass system. Appl. Therm. Eng..

[10] Delfiya A., Mohapatra D., Kotwaliwale N., Mishra A.K. (2017). Effect of microwave blanching and brine solution pretreatment on the quality of carrots dried in solar-biomass hybrid dryer. J. Food Process. Preserv..

[11] Anvari S., Khalilarya S., Zare V. (2018). Exergoeconomic and environmental analysis of a novel configuration of solar-biomass hybrid power generation system. Energy.

[12] Kang Q., Dewil R., Degrève J., Baeyens J., Zhang H. (2018). Energy analysis of a particle suspension solar combined cycle power plant. Energy Convers. Manag..

[13] Mehrpooya M., Khalili M., Sharifzadeh M.M.M. (2018). Model development and energy and exergy analysis of the biomass gasification process (Based on the various biomass sources). Renew. Sustain. Energy Rev..

[14] Ghasemi A., Heidarnejad P., Noorpoor A. (2018). A novel solar-biomass based multi-generation energy system including water desalination and liquefaction of natural gas system: Thermodynamic and thermoeconomic optimization. J. Clean. Prod..

[15] Greg P. (2018). Techno-economic comparison of the levelised cost of electricity generation from solar PV and battery storage with solar PV and combustion of bio-crude using fast pyrolysis of biomass. Energy Convers. Manag..

[16] Krarouch M., Hamdi H., Lamghari S., Outzourhit A. (2018). Simulation of floor heating in a combined solar-biomass system integrated in a public bathhouse located in Marrakech. IOP Conf. Ser. Mater. Sci. Eng..

[17] Wang J., Ying Y. (2016). Energy, exergy and environmental analysis of a hybrid combined cooling heating and power system utilizing biomass and solar energy. Energy Convers. Manag..

[18] Gustavsson L., Joelsson A., Sathre R. (2010). Life cycle primary energy use and carbon emission of an eight-storey wood-framed apartment building. Energy Build..

[19] Mclaren D. (2012). A comparative global assessment of potential negative emissions technologies. Process Saf. Environ. Prot..

[20] Xu C., Shi W., Hong J., Zhang F., Wei C. (2015). Life cycle assessment of food waste-based biogas generation. Renew. Sustain. Energy Rev..

[21] Budzianowski W.M. (2011). Can ‘negative net CO emissions’ from decarbonised biogas-to-electricity contribute to solving Poland’s carbon capture and sequestration dilemmas?. Energy.

[22] Jury C., Benetto E., Koster D., Schmitt B., Welfring J. (2010). Life Cycle Assessment of biogas production by monofermentation of energy crops and injection into the natural gas grid. Biomass Bioenergy.

[23] Distefano T.D., Belenky L.G., Safferman S., Lioa W., Saffron C. (2009). Life-cycle analysis of energy and greenhouse gas emissions from anaerobic biodegradation of municipal solid waste. J. Environ. Eng..

[24] David S., Maclean H.L., Bradley S. (2012). Electricity production from anaerobic digestion of household organic waste in Ontario: Techno-economic and GHG emission analyses. Environ. Sci. Technol..

[25] Thomassen M.A., Dalgaard R., Heijungs R., Boer I. (2008). Attributional and consequential LCA of milk production. Int. J. Life Cycle Assess..

[26] Ogunjuyigbe A.S.O., Ayodele T.R., Akinola O.A. (2016). Optimal allocation and sizing of PV/Wind/Split-diesel/Battery hybrid energy system for minimizing life cycle cost, carbon emission and dump energy of remote residential building. Appl. Energy.

[27] Nojavan S., Majidi M., Zare K. (2017). Performance improvement of a battery/PV/fuel cell/grid hybrid energy system considering load uncertainty modeling using IGDT. Energy Convers. Manag..

[28] Khare V., Nema S., Baredar P. (2016). Solar–wind hybrid renewable energy system: A review. Renew. Sustain. Energy Rev..

[29] Gan L.K., Shek J.K.H., Mueller M.A. (2015). Hybrid wind-photovoltaic-diesel-battery system sizing tool development using empirical approach, life-cycle cost and performance analysis: A case study in Scotland. Energy Convers. Manag..

[30] Lajunen A., Lipman T. (2016). Lifecycle cost assessment and carbon dioxide emissions of diesel, natural gas, hybrid electric, fuel cell hybrid and electric transit buses. Energy.

[31] Hassani S., Saidur R., Mekhilef S., Taylor R.A. (2016). Environmental and exergy benefit of nanofluid-based hybrid PV/T systems. Energy Convers. Manag..

[32] Asdrubali F., Baldinelli G., D’Alessandro F., Scrucca F. (2015). Life cycle assessment of electricity production from renewable energies: Review and results harmonization. Renew. Sustain. Energy Rev..

[33] Ozturk M., Ozek N., Batur H., Koc M. (2012). Thermodynamic and life cycle assessment of flat-plate collector, photovoltaic system and photovoltaic thermal collector. Int. J. Exergy.

[34] ISO (2006). ISO 14040 International Standard. Environmental Management–Life Cycle Assessment–Principles and Framework.

[35] Rebitzer G., Ekvall T., Frischknecht R., Hunkeler D., Norris G., Rydberg T., Schmidt W.P., Suh S., Weidema B.P., Pennington D.W. (2004). Life cycle assessment: Part 1: Framework, goal and scope definition, inventory analysis, and applications. Environ. Int..

[36] Bartolozzi I., Rizzi F., Frey M. (2017). Are district heating systems and renewable energy sources always an environmental win-win solution? A life cycle assessment case study in Tuscany, Italy. Renew. Sustain. Energy Rev..

[37] Astrup T.F., Tonini D., Turconi R., Boldrin A. (2015). Life cycle assessment of thermal Waste-to-Energy technologies: Review and recommendations. Waste Manag..

[38] Alberola-Borràs J.-A., Baker J., De Rossi F., Vidal R., Beynon D., Hooper K.E., Watson T.M., Mora-Seró I. (2018). Perovskite Photovoltaic Modules: Life Cycle Assessment of Pre-industrial. Prod. Process.

[39] Soares W.M., Athayde D.D., Nunes E.H.M. (2018). LCA study of photovoltaic systems based on different technologies. Int. J. Green Energy.

[40] Flysjö A., Henriksson M., Cederberg C., Ledgard S., Englund J.E. (2011). The impact of various parameters on the carbon footprint of milk production in New Zealand and Sweden. Agric. Syst..

[41] Battini F., Agostini A., Boulamanti A.K., Giuntoli J., Amaducci S. (2014). Mitigating the environmental impacts of milk production via anaerobic digestion of manure: Case study of a dairy farm in the Po Valley. Sci. Total Environ..

[42] Mezzullo W.G., Mcmanus M.C., Hammond G.P. (2013). Life cycle assessment of a small-scale anaerobic digestion plant from cattle waste. Appl. Energy.

[43] Styles D., Dominguez E.M., Chadwick D. (2016). Environmental balance of the UK biogas sector: An evaluation by consequential life cycle assessment. Sci. Total Environ..

[44] Ramírez Arpide R., Demirer G., Gallegos-Vázquez C., Hernández-Eugenio G., Santoyo H., Espinosa-Solares T. (2018). Life cycle assessment of biogas production through anaerobic co-digestion of nopal cladodes and dairy cow manure. J. Clean. Prod..

